# Australian gridded chloride deposition-rate dataset

**DOI:** 10.1016/j.dib.2022.108189

**Published:** 2022-04-20

**Authors:** Andy Wilkins, Russell Crosbie, Tristan Louth-Robins, Phil Davies, Matthias Raiber, Warrick Dawes, Lei Gao

**Affiliations:** a CSIRO Mineral Resources, Queensland Centre for Advanced Technologies, PO Box 883, Kenmore, Qld 4069, Australia; b CSIRO Land and Water, Locked Bag 2, Glen Osmond SA 5064, Australia; c CSIRO Land and Water, GPO Box 2583, Brisbane, Qld 4001, Australia; d CSIRO Land and Water, Private Bag 5, Wembley WA 6913, Australia

**Keywords:** Atmospheric chemistry, Chloride mass balance, Groundwater recharge, Hydrochemistry, Rainfall

## Abstract

Chloride deposition-rate measurements at points within Australia are upscaled to the entire continent on a regular 0.05° grid. The upscaling uses a double-exponential correlation between deposition rate and distance to the coast, where the parameters in the double-exponential are spatially varying. These parameters are estimated using least-squares with Tikhonov regularisation to ensure minimal spatial variability. A calibration-constrained, null-space Monte-Carlo analysis is used to quantify uncertainty in the prediction. The resulting dataset consists of the best-fit chloride deposition rates across Australia, as well as estimates of uncertainty. The dataset can be used for various purposes including: estimating groundwater recharge through the use of the chloride mass-balance method; catchment salt balance estimates; regional investigations of groundwater hydrochemistry; and, corrosion prediction.

## Specifications Table

**Table:** 

Subject	Environmental Science: Hydrology and Water quality
Specific subject area	Chloride deposition rate in Australia
Type of data	Tables, Rasters, computer scripts
How the data were acquired	Raw data acquired from a literature review of published and unpublished sources listed in Table 1. The upscaling to gridded chloride deposition-rates across Australia was performed using the PEST software along with the python pykrige package and custom python scripts included with the data.
Data format	Analysed
Description of data collection	Source chloride deposition data from the literature review were weighted with 1.0 if at least one year of continuous collection of rainfall with well-documented methods; 0.8 is reliable but not published; 0.6 less than full year of data collection but all rainfall collected; 0.4 not fully documented; 0.2 less than full year, not all rainfall collected; 0 otherwise. Output rasters are summaries of 1000 replicates.
Data source location	See Table 1
Data accessibility	Repository name: Australian chloride deposition rate (1937-2021) Data identification number: v1 Direct URL to data: https://doi.org/10.25919/zkr0-fw05

## Value of the Data

• Chloride deposition rates have been measured at 367 isolated locations across the continent but this is not of sufficient density for many purposes. This dataset upscales these data to a regular gridded deposition rate with its associated uncertainty that can be interrogated for all points in Australia, making it useful for studies outside the 367 isolated locations.
• Hydrologists, hydrogeologists and hydrogeochemists will find this dataset useful for defining a baseline chloride input to the landscape at point, catchment or continental scale.
• These data can be used to estimate groundwater recharge through the chloride mass-balance method, are useful in catchment salt balance studies, investigations of groundwater hydrochemical evolution and can be used for predicting corrosion.

## 1 Data Description

### 1.1 Raw (primary data)

observations_update_v04.csv. Plaintext CSV file with columns labelled Table 1:

**Table 1:** Data sources.

Data sources G.P. Ayers, The Chemical Composition of Precipitation: A Southern Hemisphere Prospective, in: Atmospheric Chemistry. Edited by E.D. Goldberg. Berlin: Springer, 1982. E. B. Bekele, R. B. Salama, D. P. Commander, C. J. Otto, W. P. Hick, G. D. Watson, D. W. Pollock, P. A. Lambert, Estimation of Groundwater Recharge to the Parmelia Aquifer in the Northern Perth Basin 2001-2002. CSIRO Land and Water Technical Report 10/03, 2003. E. Bettenay, A. V. Blackmore, F. J. Hingston. Aspects of the Hydrologic Cycle and Related Salinity in the Belka Valley, Western Australia, Australian Journal of Soil Research 2, no. 2 (1964): 187-210. https://doi.org/10.1071/SR9640187. A. J. W. Biggs, Rainfall Salt Accessions in the Queensland Murray-Darling Basin, Soil Research 44, no. 6 (2006): 637-45. https://doi.org/10.1071/SR06006. G. Blackburn, S. McLeod, Salinity of Atmospheric Precipitation in the Murray-Darling Drainage Division, Australia, Australian Journal of Soil Research 21, no. 4 (1983): 411-34. https://ejournal.csiro.au/cgi-bin/sciserv.pl?collection=journals&journal=00049573&issue=v21i0004&article=411_soapitmdda. M. E. Bormann, "Temporal and Spatial Trends in Rainwater Chemistry across Central and Western Victoria. Bsc Honours Thesis, 2004. R. S. Crosbie, The Regional Scaling of Groundwater Recharge, PhD Thesis, University of Newcastle, 2003. R. S. Crosbie, D. Morrow, R. Cresswell, F. Leaney, S. Lamontagne, M. Lefournour, New Insights to the Chemical and Isotopic Composition of Rainfall across Australia. CSIRO Water for a Healthy Country Flagship, Australia, 2012. I. Douglas, The Effects of Precipitation Chemistry and Catchment Area Lithology on the Quality of River Water in Selected Catchments in Eastern Australia, Earth Science Journal 2, no. 2 (1968): 128-44. P. Farrington, G. A. Bartel, Accession of Chloride from Rainfall on the Gnangara Groundwater Mound, Western Australia, CSIRO Technical Memorandum 88/1, 1988. P. Farrington, R. B. Salama, G. A. Bartle, G. D. Watson, Accession of Major Ions in Rainfall in the South Western Region of Western Australia, CSIRO Divisional Report 93/1, 1993. J. N. Galloway, G. E. Likens, W. C. Keene, J. M. Miller, The Composition of Precipitation in Remote Areas of the World, Journal of Geophysical Research 87, no. C11 (1982): 8771–86. H. Guan, A. J. Love, C. T. Simmons, O. Makhnin, A. S. Kayaalp, Factors Influencing Chloride Deposition in a Coastal Hilly Area and Application to Chloride Deposition Mapping, Hydrol. Earth Syst. Sci. 14, no. 5 (2010): 801-13. http://www.hydrol-earth-syst-sci.net/14/801/2010/ F. J. Hingston, V. Gailitis, The Geographic Variation of Salt Precipitated over Western Australia, Australian Journal of Soil Research 14, no. 3 (1976): 319-35. J. T. Hutton, Rainwater Analysis: July 1957 to March 1961, CSIRO Divisional Report 7/62, 1962. J. T. Hutton, Rainwater Analysis, CSIRO Divisional Report 8/57, 1957. J. T. Hutton, T. I. Leslie, Accession of Non-Nitrogenous Ions Dissolved in Rainwater to Soils in Victoria, Article, Australian Journal of Agricultural Research 9, no. 4 (1958): 492-507. M. Keywood, Origins and Sources of Atmospheric Precipitation from Australia: Chlorine-36 and Major-Element Chemistry, Australian National University, 1995. P. J. Langkamp, M. J. Dalling, Nutrient Cycling in a Stand of Acacia Holosericea, A. Cunn. Ex G. Don. Iii. Calcium, Magnesium, Sodium and Potassium, J Australian Journal of Botany 31, no. 2 (1983): 141-49. https://doi.org/10.1071/BT9830141. G. E. Likens, W. C. Keene, J. M. Miller, J. M. Galloway, Chemistry of Precipitation from a Remote, Terrestrial Site in Australia, Journal of Geophysical Research: Atmospheres 92, no. D11 (1987): 13299-314.
J. L. Martinez, M. Raiber, M. E. Cox, Assessment of Groundwater–Surface Water Interaction Using Long-Term Hydrochemical Data and Isotope Hydrology: Headwaters of the Condamine River, Southeast Queensland, Australia, Science of The Total Environment 536 (2015): 499-516. https://doi.org/10.1016/j.scitotenv.2015.07.031. B. N. Noller, N. A. Currey, G. P. Ayers, R. W. Gillett, Chemical Composition and Acidity of Rainfall in the Alligator Rivers Region, Northern Territory, Australia, Science of The Total Environment 91 (1990): 23-48. M. E. Probert, The Composition of Rainwater at Two Sites near Townsville, Qld, Australian Journal of Soil Research 14, no. 3 (1976): 397-402. M. Raiber, J. A. Webb, D. A. Bennetts, Strontium Isotopes as Tracers to Delineate Aquifer Interactions and the Influence of Rainfall in the Basalt Plains of Southeastern Australia, Journal of Hydrology 367, no. 3 (2009): 188-99, https://doi.org/10.1016/j.jhydrol.2008.12.020. T.R. Ransley, Personal communication, Unpublished data. M. E. Sweeney, C. L. Moore, K. G. McQueen, T. Spandler, Geomorphic Controls on Deposition of Salt in the Greater Tamar Catchment, Northeast Tasmania, Australian Journal of Earth Sciences (2016): 1-12. https://doi.org/10.1080/08120099.2016.1212400. L. J. H. Teakle, The Salt (Sodium Chloride) Content of Rainwater, J. Agric. W. Aust. 14 (1937): 115-23. J. Turner, M. J. Lambert, J. Knott, Nutrient Inputs from Rainfall in New South Wales State Forests, Forest Research and Development Division, State Forests of New South Wales, 1996. R. Wetselaar, J. T. Hutton, The Ionic Composition of Rainwater at Katherine, NT. and Its Part in the Cycling of Plant Nutrients, Australian Journal of Agricultural Research 14, no. 3 (1963): 319-29. https://doi.org/10.1071/AR9630319. D. Wilson, P. G. Cook, L. B. Hutley, S. Tickell, P. Jolly, Effect of Land Use on Estimates of Evapotransporation and Recharge in the Daly River Catchment, Department of Natural Resources, Environment and the Arts, Technical Report No. 17/2006D, 2006.

SITE_NO (our unique identifier for this raw data)

LONGITUDE (degrees, GDA94)

LATITUDE (degrees, GDA94)

CHLORIDE_OBS (chloride deposition-rate observation, in kg/hectare/year)

WEIGHT (the weight prescribed to the observation: a measure of reliability)

DIST_TO_COAST (distance to coast of observation, in metres)

REFERENCE (the source from which the data was extracted)

observations_update_v04.png: Graphical representation of the measured chloride deposition rates found in observations_update_v04.csv

### 1.2 Output data (secondary data)

mean.tif: GeoTiff file in the GDA94 coordinate system describing the mean chloride deposition across Australia, with units kg/hectare/year. This is defined to be 10^(mean(log10(chloride deposition))), where the mean is taken over 1000 uncertainty-run replicates. This formula is used because the uncertainty analysis is formulated in log10 units.

sd.tif: GeoTiff file in the GDA94 coordinate system describing the standard deviation of chloride deposition from 1000 replicates of chloride deposition-rate across Australia.

sdlog10.tif: GeoTiff file in the GDA94 coordinate system describing the standard deviation of log10 of chloride deposition from 1000 replicates of chloride deposition-rate across Australia.

skew.tif: GeoTiff file in the GDA94 coordinate system describing the skewness of chloride deposition from 1000 replicates of chloride deposition-rate across Australia.

skewlog10.tif: GeoTiff file in the GDA94 coordinate system describing the skewness of log10 of chloride deposition from 1000 replicates of chloride deposition-rate across Australia.

5th_percentile.tif: GeoTiff file in the GDA94 coordinate system describing the 5th percentile of chloride deposition across Australia. This is defined to be 10^(5th percentile of log10(chloride deposition))

95th_percentile.tif: GeoTiff file in the GDA94 coordinate system describing the 95th percentile of chloride deposition across Australia. This is defined to be 10^(95th percentile of log10(chloride deposition))

uncertainty_results.png: Graphical representation of: mean chloride deposition, standard deviation of chloride deposition, skewness of chloride deposition, skewness of log10 deposition, 5th percentile of deposition and 95th percentile of deposition. The GeoTiff data is machine readable, while this file is more easily interpretable by humans.

### 1.3 Computer scripts (see methods section for detailed explanations)

pilot_to_obs.py: python3 script that uses kriging to interpolate the values of A1, A2, l1 and l2 (see Methods section) in the pilot-point file to observation points. PEST treats this as ``the model'' and runs it many times during iterating to the best solution for A1, A2, l1 and l2. PEST also uses it during the uncertainty analysis.

randpar_1.py: python3 script that generates the PEST ``par'' files used in the PEST uncertainty analysis.

generate_pest_files.py: python3 script that uses the observations and the pilot points to write PEST tpl, pst, ins files as well as the windows batch file to run the ``model'' pilot_to_obs.py.

run_uncertainty.bat: windows batch file to run the null-space Monte-Carlo uncertainty analysis.pilot_to_obs.py: python3 script that perform

## 2 Experimental Design, Materials and Methods

### 2.1 Background

Chloride deposition rates have been collated from measurements at 367 sites across Australia. These data are compiled into observations_update_v04.csv and shown graphically in observations_update_v04.png. The data sources are listed in Table 1. These measurements are of bulk rainfall and contain both wet and dry deposition of chloride.

Davies and Crosbie [1] describe a method for extrapolating such chloride deposition data to the entire continent. Here, their method is explained in more detail than the original paper and used to estimate chloride deposition across the continent. Davies and Crosbie [1] used 291 observations in their calibration of the continental scale model and an additional 20 observations as validation sites. Here, a comprehensive set of source data consisting of 367 observations is used, this raw dataset has been updated using data collated 10 years after Davies and Crosbie [1] collated their data in 2010.

### 2.2 Weighting the observations

Each observation is provided with a weight of between 0 and 1, $w_{p}$, depending on its relative quality of field measurements, which is also listed in the raw data observations_update_v04.csv. It is generally assumed [2], [3], [4] that a complete year of data is the minimum time period required to obtain a representative estimate of the chloride deposition. The weight for each observation was chosen using the following method:

• 1.0: most reliable data available, at least one year of continuous collection of rainfall with well documented methods
• 0.8: reliable source of data but not published
• 0.6: less than full year, but collected all rainfall (typically tropics)
• 0.4: reliable source of data but not fully documented
• 0.2: less than full year, but did not collect all rainfall
• 0: not used in calibration, data of very poor quality

**Fig. 1:**
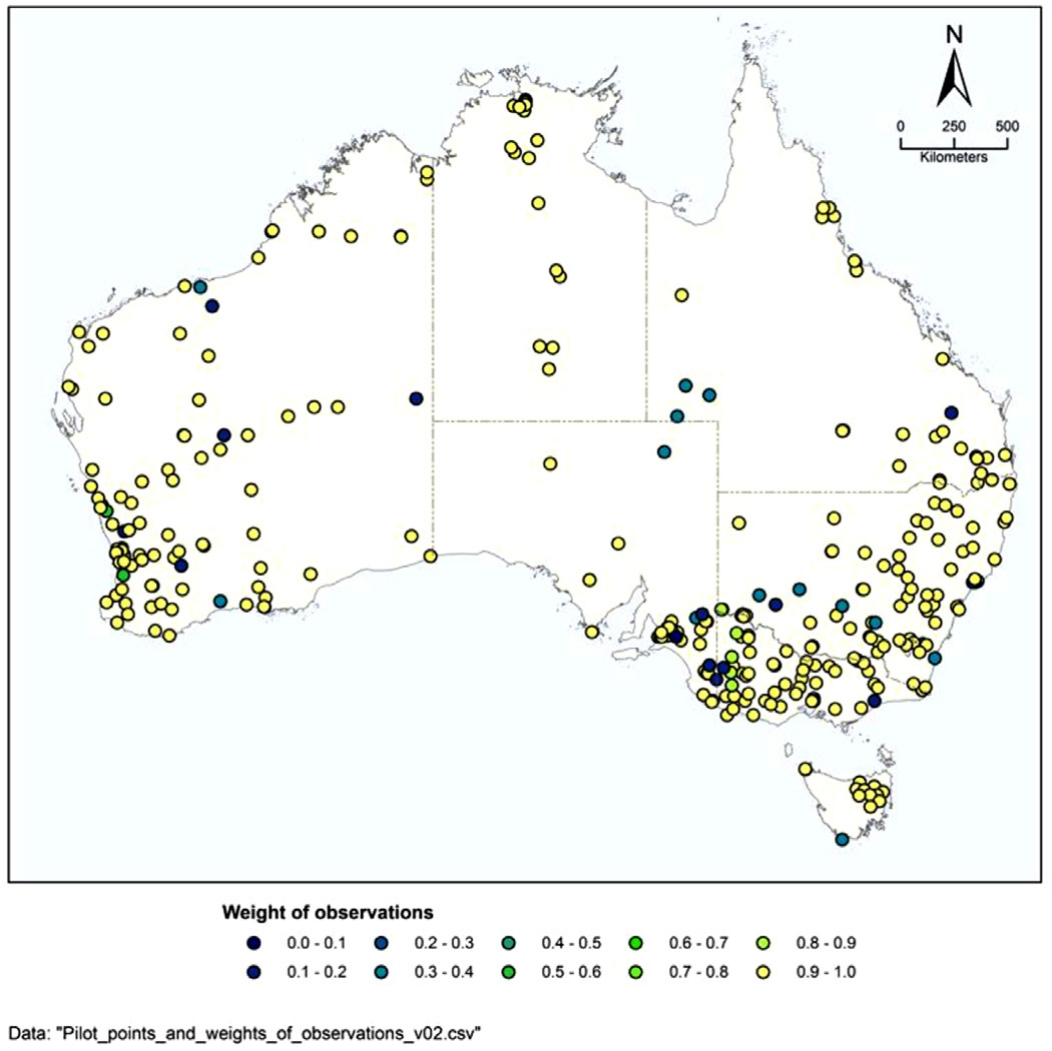
Location and weight of each chloride deposition observation.

The result is shown in Fig. 1.

### 2.3 Relationship with distance to coast

Davies and Crosbie [1] explored correlations between the observed deposition rates and distance to the coast, elevation, terrain slope, difference in angle between the aspect of the slope and direction to the coast, mean annual rainfall and mean annual windspeed. The only strong correlation was between deposition and distance to the coast. Based on work by Keywood [5], they postulated a relationship of the form:

(1) $$D=A_{1}e^{-\frac{d}{\lambda_{1}}}+A_{2}e^{-\frac{d}{\lambda_{2}}}$$

Here:

• D is the deposition rate, with units kg.ha^−1^.yr^−1^;
• d is the distance to the coast, with units km;
• $A_{1}\;$is a coefficient, with units kg.ha^−1^.yr^−1^;
• $A_{2}\;$is a coefficient, with units kg.ha^−1^.yr^−1^;
• $\lambda_{1}$ is a decay constant, with units km;
• $\lambda_{2}$ is a decay constant, with units km.

The physical motivation for this form is that there are two sources of deposition as described by Keywood [5]:

• the “fast” component, $A_{1}e^{-\frac{d}{\lambda_{1}}}$, which represents the wet and dry deposition in aerosols sourced from the ocean;
• the “slow” component, $A_{2}e^{-\frac{d}{\lambda_{2}}}$, which represents the deposition of gaseous chloride that is formed by volatilisation of chloride in sea salt at pH less than 3.

**Fig. 2:**
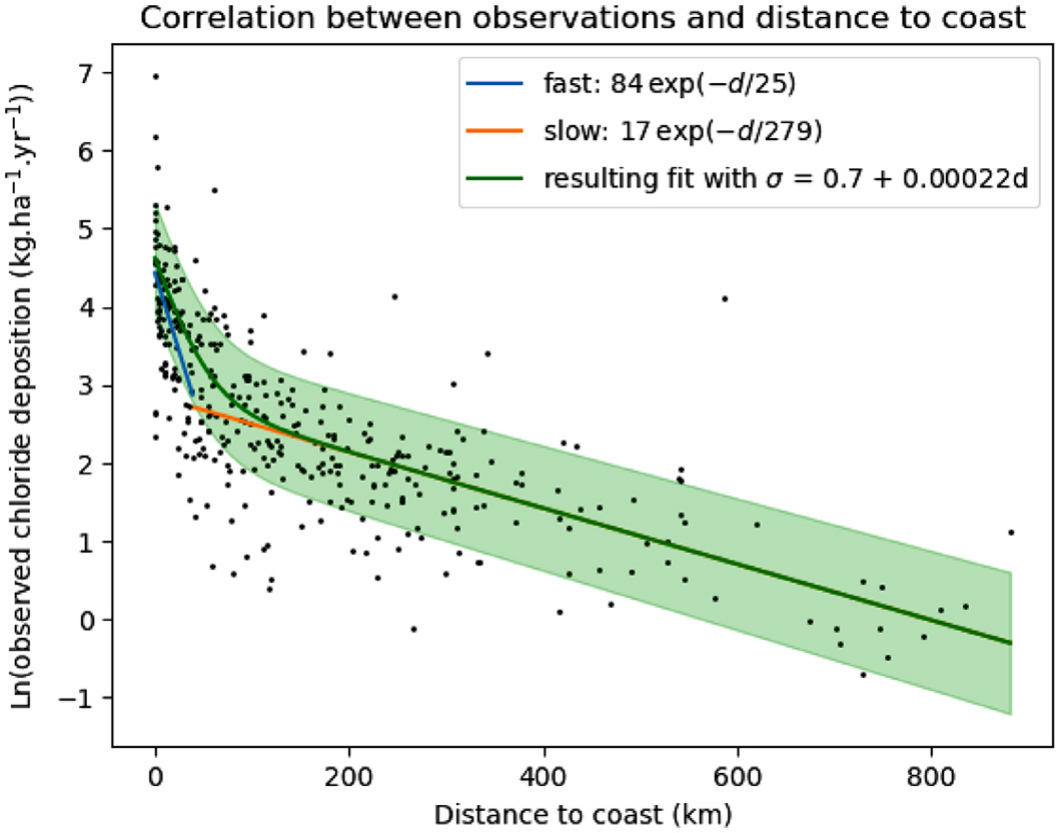
Correlation between the observed chloride deposition and the distance to the coast. The blue and orange lines are fits to the near-coast and central-region data, respectively. The green line is the resulting fit from Eq. (1). The shaded region and σ is described below.

This correlation is also clear in the 367 observations considered here, as shown in Fig. 2, where a least-squares fit results in:

• $A_{1}=84\;$kg.ha^−1^.yr^−1^
• $A_{2}=17\;$kg.ha^−1^.yr^−1^
• $\lambda_{1}=25\;$km
• $\lambda_{2}=279$ km

The least-squares fits are performed to provide a suitable initialisation for the PEST algorithms described below, they are not used in any other analyses.

### 2.4 Scatter of repeat measurements

The Monte-Carlo uncertainty analysis described below requires uncertainty to be quantified in one form or another (more specifically, the prior or posterior uncertainty distribution can be quantified). One way of estimating uncertainty is by exploring data from observation sites where repeated measurements have been performed. If, for example, the observations remain unchanged with time, then it is reasonable to infer that the observations are highly reliable, which means the prediction will be more reliable than the case where the observations at each site fluctuate wildly.

It is generally assumed [2], [3], [4] that a complete year of data is the minimum time period required to obtain a representative estimate of the chloride deposition, and the amount of chloride deposition is not considered dependent upon the amount of rainfall. However, Davies and Crosbie [1] found that there is some dependence on rainfall amount.

Katherine in the NT is the best example in the dataset of temporal fluctuations in chloride deposition, and that using a single year of observation can result in considerable uncertainty. In Katherine, there have been 5 independent studies measuring the chloride deposition spread over five decades and ranging from 1 to 5 years duration. These studies have reported a chloride deposition rate of between 2.46 to 7.30 kg.ha^−1^.yr^−1^ [5], [6], [7], [8], [9].

**Fig. 3:**
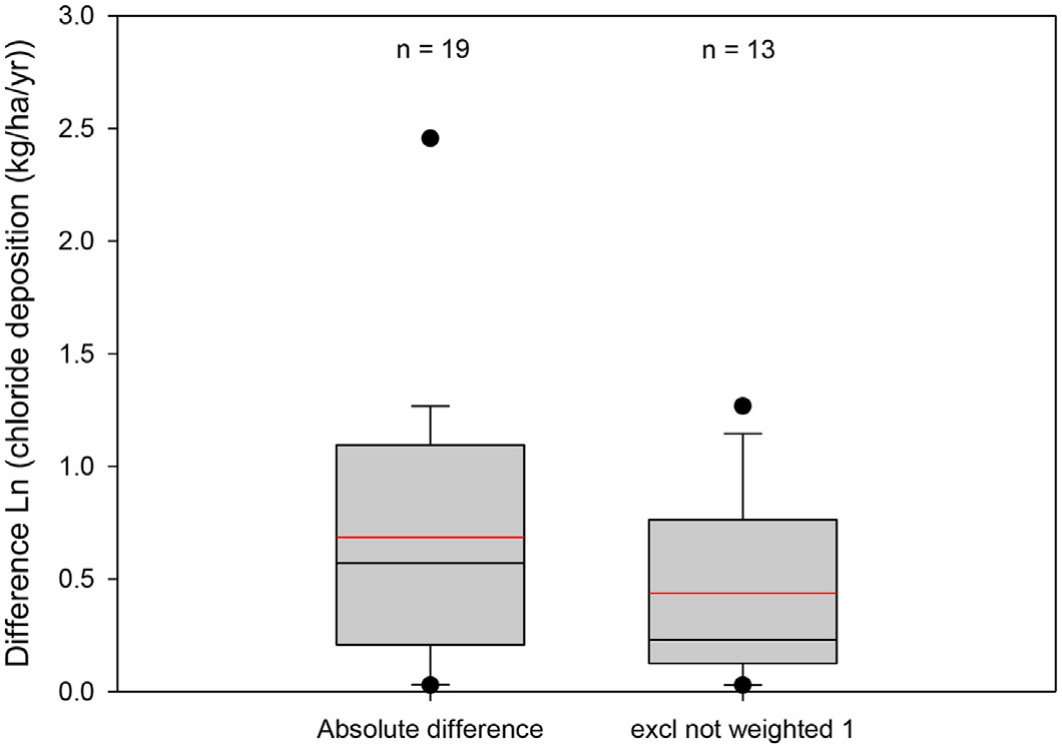
Boxplots of the difference in chloride deposition measured at the same site by difference studies. (Red line is the mean of the absolute difference in the natural log transformed observations).

In the dataset collated here, there are 19 sites that have repeat measurements. The difference in the observations is a measure of the uncertainty in the chloride deposition within the observed dataset. The mean difference of the natural-log transformed data is 0.68, if the observations that do not have a weight of 1 are excluded, this decreases to 0.44, as shown in Fig. 3.

### 2.5 Scatter of observations around distance-to-coast formula

**Fig. 4:**
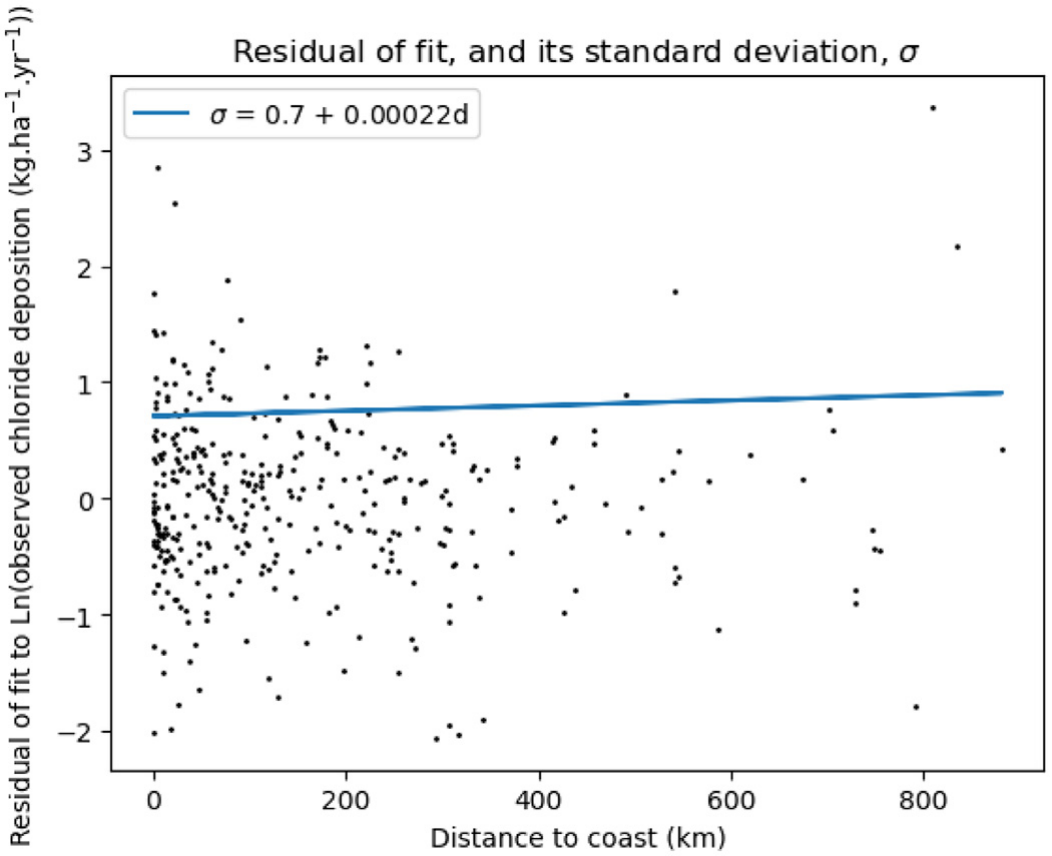
Scattered points: the residual, which is the difference between the observation and lnD of Eq. (1). The standard deviation, σ, of this residual may be fitted with a straight line: σ=0.7+0.00022d.

Another way of estimating the uncertainty of the prediction is to explore the scatter of the observations around the model given by Eq. (1). The difference between lnD of Eq. (1) and the logarithm of the observations is called the residual, which is plotted in Fig. 4. The scatter is approximately normally-distributed, and the standard deviation, σ, of the residuals may be computed, which results in:

σ=0.7+0.00022d

where d is measured in km (this is also shown in Fig. 4, and assumes that D is measured in kg.ha^−1^.yr^−1^). The standard deviation, σ, is only weakly dependent on distance from the coast, which may reflect that experimental error is virtually independent of where the sample is performed, or may reflect that the model of Eq. (1) is equally valid at all points. Using this value to perturb the fit of lnD expressed through Eq. (1) results in the green shaded region of Fig. 2.

### 2.6 Pilot points and kriging scheme

The method of Davies and Crosbie [1] uses the PEST software [10] to estimate $A_{1},A_{2},\lambda_{1},\lambda_{2}$ at “pilot points” scattered throughout Australia, and to interpolate these to the remainder of Australia using kriging. The PEST process is described in sections below: this section describes the pilot points and kriging.

**Fig. 5:**
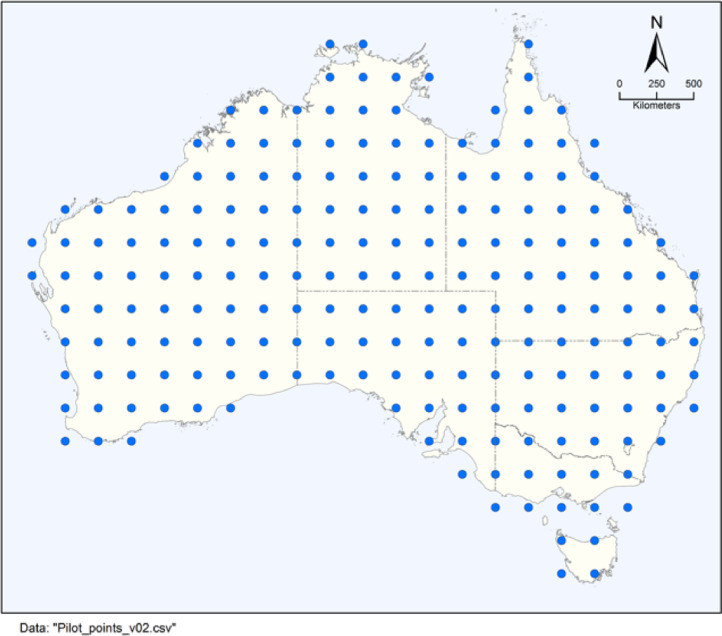
Pilot points used in this study.

An evenly spaced grid of pilot points is used here, with spacing being 2 degrees in latitude and longitude, as shown in Fig. 5.

Ordinary kriging is used to interpolate $A_{1},A_{2},\lambda_{1},\lambda_{2}$ from the pilot points to a finer grid of 0.05 degrees (∼5 km) across Australia. The python PyKrige software package [11] is employed. The key ingredient to the kriging process is the variogram. In keeping with Davies and Crosbie [1], an exponential variogram is used here:

$$v=p\left(1-e^{-\frac{\hat{d}}{r}}\right)+n$$

where

• p is the partial sill, with value 100;
• $\hat{d}$ is the distance between points, measured in degrees;
• r is the range, with value 120 deg;
• n is the nugget, with value 1.

**Fig. 6:**
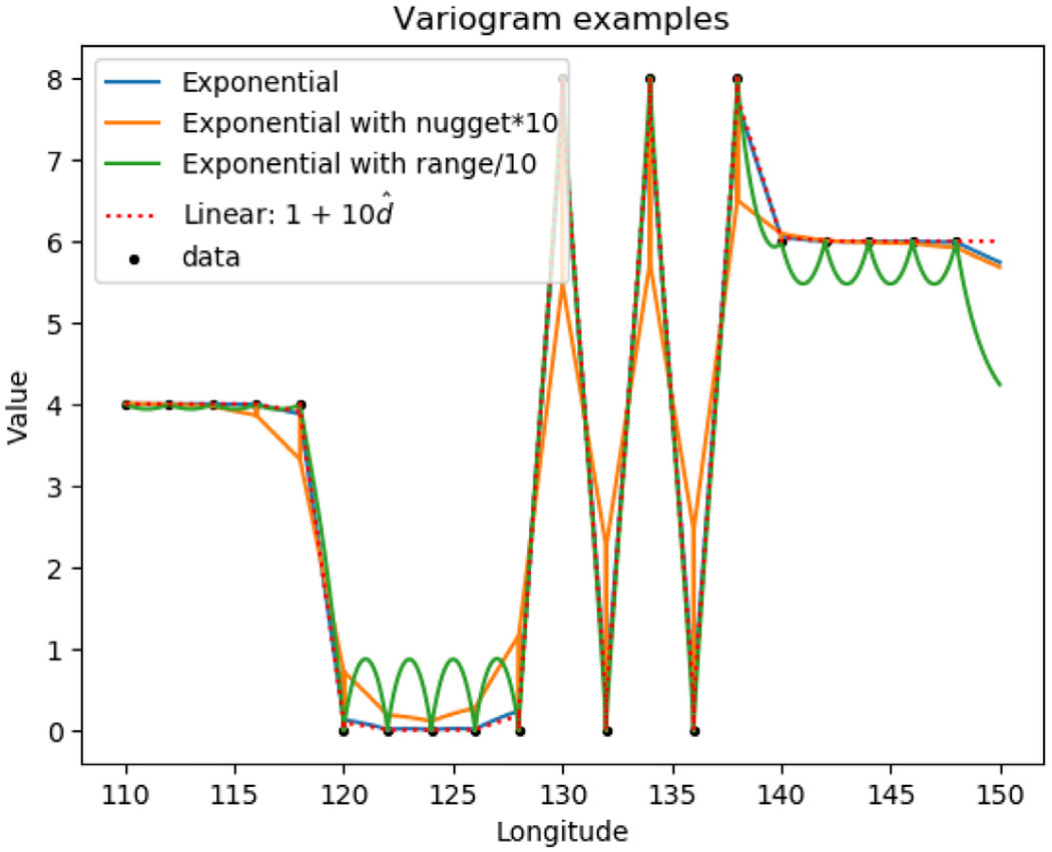
Kriging some artificial data (black dots) using 4 different variograms.

Some example results are shown in Fig. 6. Only certain aspects of the variogram are important (for example, the overall scaling is irrelevant to the result) and different variograms can produce similar results (compare the base “exponential” type with the linear variogram of the form $v=1+10\hat{d}$ in Fig. 6). The final chloride-deposition results are not strongly dependent on the form of the variogram.

### 2.7 Core PEST process

The PEST software [10] is used to find the best values (in a least-squares sense) of $A_{1},A_{2},\lambda_{1},\lambda_{2}$ at each pilot point. Since there are 205 pilot points, this means the minimisation involves 820 unknowns. Each iteration of the PEST process involves the following steps.

1. Initial values for the 820 unknowns are provided are provided from Fig. 2.
2. The kriging procedure described above is used to find $A_{1},A_{2},\lambda_{1},\lambda_{2}$ at each observation point.
3. Eq. (1) provides the predicted value for chloride deposition at each observation point.
4. The objective function, J, which quantifies the differences between these predictions and each observation, is computed:
  $$J=\sum_{p}w_{p}^{2}{\left(\log_{10}\left(\text{Predictio}n_{p}\right)-\log_{10}\left(\text{Observatio}n_{p}\right)\right)}^{2}$$

where p indicates the observation point, and $w_{p}$ is the weight of each observation. Note that log_10_ is used here, in contrast to ln used above.

5. The Jacobian, which is the change of the objective function with respect to changes in $A_{1},A_{2},\lambda_{1},\lambda_{2}$ at each pilot point, is calculated. This is used to supply new values for the 820 unknowns such that J will decrease. The process then starts again from Step (2) using these new values.

In the simplest case, PEST minimises the error, J, to provide the best values of $A_{1},A_{2},\lambda_{1},\lambda_{2}$. However, given the number of unknowns is greater than the number of observations, this simple model is likely to suffer from overfitting, and PEST can quite easily find solutions with small J. The overfitting is eliminated by using two additional ingredients.

Firstly, a target J is provided to PEST to define when the iterative procedure described above should stop. Above, the uncertainty inherent within the observations was found to be approximately 0.44 on a natural-log scale. Assuming these uncertainties are representative of an acceptable mismatch between the model and the data, then for 367 observations a reasonable target is $J_{\text{target}}\approx 367\;\times {\left(\frac{0.44}{\ln10}\right)}^{2}\approx 13.5$. This alone does not eliminate overfitting: instead, it simply provides PEST freedom to find multiple solutions.

To find the solution that agrees most strongly with the model in Eq. (1), a second ingredient is used: PEST allows weak constraints to be placed on the unknowns. This feature is used to ensure that $A_{1},A_{2},\lambda_{1},\lambda_{2}$ are similar at nearby pilot points. This means that the parameters determining the chloride deposition prediction will “vary smoothly” over the continent. Specifically, the constraints take the form of

$$C_{ij}^{A_{1}}=\log_{10}\left(A_{1}^{i}\right)-\log_{10}\left(A_{1}^{j}\right)=0\;\text{with}\;\text{weight}\;w_{ij}$$

and similarly for $A_{2},\lambda_{1},\lambda_{2}$. Here the superscripts i and j indicate pilot points. Here, given the distance $\hat{d}$ (in degrees) between two pilot points, the weights are $w=10/\sqrt{\hat{d}}$ for $A_{1}$and $A_{2}$ if $\hat{d}\leq 5\text{deg}$, and zero otherwise. These are similar to Davies and Crosbie [1], who used approximately: $w=5.3/{\hat{d}}^{0.31}$ for $A_{1}$and $A_{2}$; $w=6.6/{\hat{d}}^{0.35}$ for $\lambda_{1}$; and $w=7.5/{\hat{d}}^{0.3}$ for $\lambda_{2}$. PEST forms the “regularisation objective function” and uses the Tikhonov process to minimise this as well as J.

## 3 Calibration-constrained Null-space Monte Carlo Approach

Following Davies and Crosbie [1], a calibration-constrained null-space Monte Carlo analysis is used to estimate the uncertainty in the PEST prediction. The idea is that while PEST has provided a set of $A_{1},A_{2},\lambda_{1},\lambda_{2}$ that minimise the error expressed through J and the constraints, there may be many other parameter combinations that are just as good. The procedure is called a “calibration-constrained null-space Monte Carlo analysis” [12] involves identifying many model scenarios that are equally well validated (i.e., also have small J), and is explained in detail below. All these model scenarios are derived from a stochastic process (Monte Carlo) and after subsequent adaptations, they all provide an acceptable fit to the historical observations.

This procedure is also performed by PEST, using the following steps:

1. Pre-calculation step.
  a. $A_{1},A_{2},\lambda_{1},\lambda_{2}$ are set to their best-fit values found by PEST in the previous step, the constraints removed, and the Jacobian is calculated by PEST. The Jacobian provides an estimate of the change in J as each of the parameters ($A_{1},A_{2},\lambda_{1},\lambda_{2}$ at the pilot points) is varied individually.
  b. PEST is used to find the “null-space”. These are certain combinations of $A_{1},A_{2},\lambda_{1},\lambda_{2}$ that do not increase J “too much”. In this case, the optimisation procedure described above resulted in J=13.5 at the optimal solution. Here, “too much” is defined to be J≤15.5. For example, it may be found that varying $A_{1}$ at a pilot point in central Queensland does not increase J by too much, so this $A_{1}$ is part of the null space. Of course, changing this $A_{1}$may impact the prediction of chloride deposition in Queensland, but it does not impact the agreement with the observations too much.
  c. In the case at hand, PEST finds that the solution space contains only about 130 independent parameters: most of the 820 parameters are actually in the null space and do not impact the result too much.
2. Null-space Monte Carlo step. 1000 random parameter sets are generated. Recall from Fig. 3 that the uncertainty in observations is roughly 0.44 or 0.68, and that from Fig. 4, the scatter is 0.7. These values provide a rough idea of the size of desired variations in lnD in the random parameter sets.
  a. Monte Carlo process: The 1000 sets are generated starting from the best-fit values mentioned in Step (1). Then, at *all* pilot points, the values of $A_{1},A_{2},\lambda_{1},\lambda_{2}$ are randomly perturbed using the following procedure:
    i. For each pilot point, a random number, z, is chosen from a normal distribution with zero mean and standard deviation σ=0.4(suggested from Figs. 3 and 4). The value of z is added to lnD given by Eq. (1), that is $\ln D^{new}=\ln\left(D\right)+z$. To implement this $A_{1},A_{2},\lambda_{1},\lambda_{2}$ at the pilot point in question are altered. Of course, the changes in the individual values $A_{1},A_{2},\lambda_{1},\lambda_{2}$ are not unique, for instance only $A_{1}$ could be changed, leaving the remaining quantities fixed. The following steps define the random procedure for perturbing $A_{1},A_{2},\lambda_{1},\lambda_{2}$.
    ii. Define $r=\frac{A_{1}\text{exp}\left(-\frac{d}{\lambda_{1}}\right)}{A_{1}\exp\left(-\frac{d}{\lambda_{1}}\right)+A_{2}\text{exp}\left(-\frac{d}{\lambda_{2}}\right)}$. Choose another random number $z_{r}$ from a uniform distribution with minimum 0 and maximum r. Then the “fast” term is perturbed by $z_{r}z,$ while the “slow” term is perturbed by $(1-z_{r})z$. That is, $\ln\left(A_{1}^{new}\right)-\frac{d}{\lambda_{1}^{new}}=\ln\left(A_{1}\right)-\frac{d}{\lambda_{1}}+z_{r}z$, and similarly for the “slow” term. This means, for instance, if the “fast” term is much smaller than the total (such as in central Australia, so r≪1) then its perturbation is small. Similarly, if the “slow” term is much smaller than the total (such as near the coast, so r∼1) then its perturbation is small. This avoids perturbing terms that do not greatly impact the chloride deposition prediction. The result is approximately $\ln D^{new}\approx \ln\left(D\right)+z$. To quantify the “approximately”: the process described here produces perturbations of lnD that are normally distributed with standard deviation 0.35 instead of 0.4, which is reflected in the results.
    iii. Consider the “fast” term. The quotient $z_{r}z/\text{ln}\left(A_{1}\right)$ is the fractional perturbation desired in $A_{1}$, if $\lambda_{1}$ were kept fixed. This gives an estimate for the perturbations required. Choose another random number, $z_{1}$, from a uniform distribution with minimum $-z_{r}z/\text{ln}\left(A_{1}\right)$ and maximum $z_{r}z/\text{ln}\left(A_{1}\right)$, and set $\lambda_{1}^{new}=\lambda_{1}\left(1+z_{1}\right)$. Now set $A_{1}^{new}$ such that $\ln\left(A_{1}^{new}\right)-d/\lambda_{1}^{new}=\ln\left(A_{1}\right)-d/\lambda_{1}+z_{r}z$.
    iv. The same method is used for the “slow” term (substitute “2” for “1” in the above).
  b. Null-space process: Any random perturbations that are not part of the null space are zeroed. For instance, random perturbations of the $A_{1}$mentioned in Step (1b) would be retained, since it is part of the null space. On the other hand, if $\lambda_{1}$at a certain pilot point in Western Australia was not part of the null space (i.e., it does impact the agreement with observations) then its random perturbations would be zeroed, so it would assume its best-fit value in each of the 1000 parameter sets.

The result of is 1000 parameter sets that should agree with observations just as well as the original of Step (1). However, because of nonlinearities, perfect agreement is rare: the mean of J over the 1000 parameter sets is $\bar{J}=13.6$ (and the standard deviation is 0.07). Because the Monte-Carlo process independently alters of $A_{1},A_{2},\lambda_{1},\lambda_{2}$ at each pilot point, the values of R are substantially higher than the best-fit value of around 250: the mean of R over the 1000 parameter sets is $\bar{R}=1600$ (and the standard deviation is 190).

**Fig. 7:**
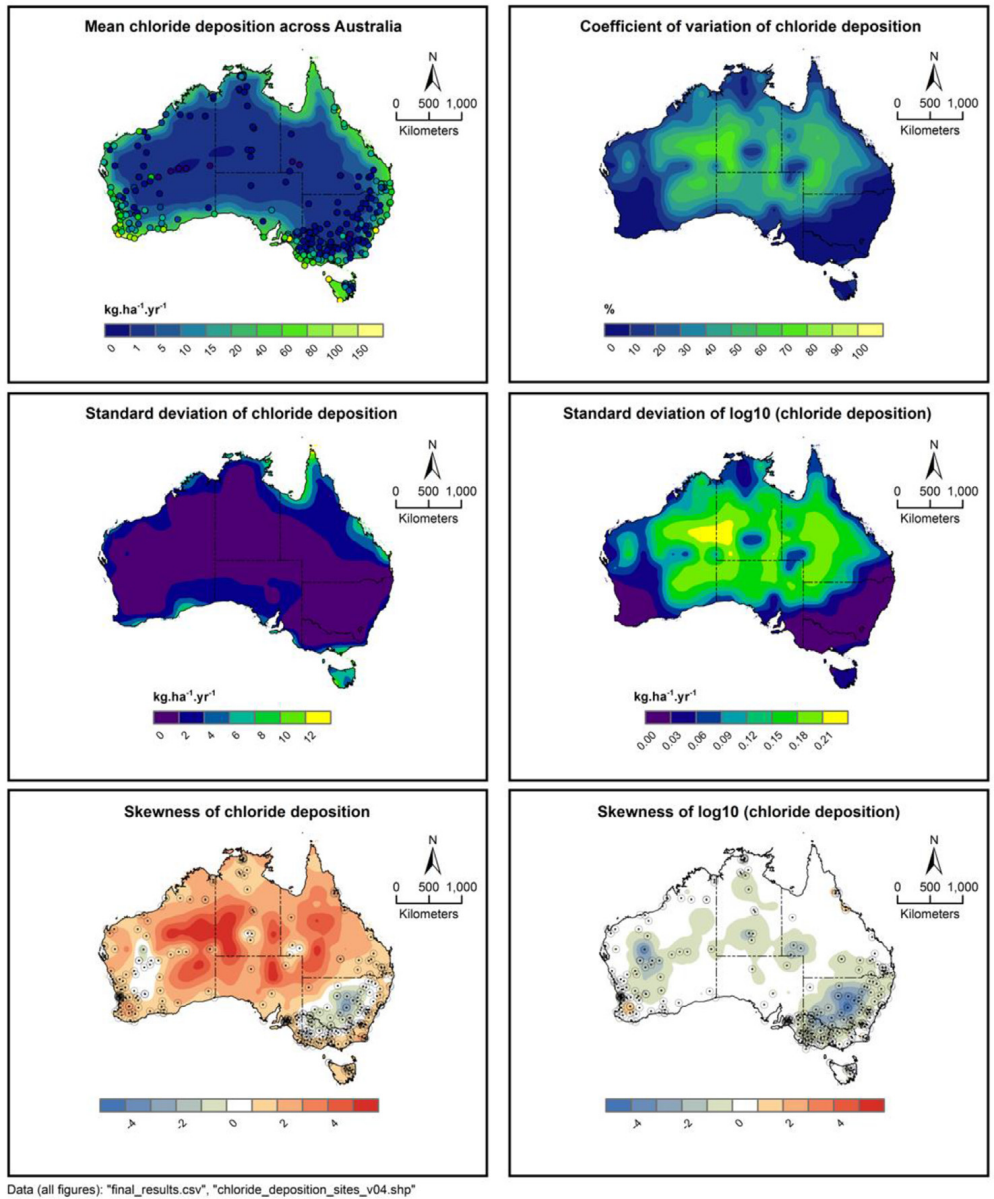
Results of the uncertainty analysis.

Using kriging described above, each of these 1000 sets can be used to provide a prediction of chloride deposition over Australia. At each point in Australia, the mean, and various other statistics of these 1000 predictions may be calculated. This yields the output datasets, shown graphically in Fig. 7.

## 4 Contextual Summary

Hydrologists, hydrogeologists and hydrogeochemists will find the resulting datasets useful for defining a baseline chloride input to the landscape at point, catchment or continental scale. The results can be used to estimate groundwater recharge through the chloride mass-balance method, are useful in catchment salt balance studies, investigations of groundwater hydrochemical evolution from recharge areas to deeper parts of aquifers and can be used for predicting corrosion of infrastructure assets.

## CRediT authorship contribution statement

**Andy Wilkins:** Methodology, Software. **Russell Crosbie:** Conceptualization, Methodology, Supervision. **Tristan Louth-Robins:** Data curation, Writing – original draft, Visualization. **Phil Davies:** Methodology, Resources. **Matthias Raiber:** Investigation, Resources. **Warrick Dawes:** Writing – original draft. **Lei Gao:** Writing – original draft.

## Declaration of Competing Interest

The authors declare that they have no known competing financial interests or personal relationships that could have appeared to influence the work reported in this paper.

## Data Availability

Australian chloride deposition rate (1937-2021) (Original data) (CSIRO Data Access Portal).
